# Reference genes for proximal femoral epiphysiolysis expression studies in broilers cartilage

**DOI:** 10.1371/journal.pone.0238189

**Published:** 2020-08-25

**Authors:** Ludmila Mudri Hul, Adriana Mércia Guaratini Ibelli, Jane de Oliveira Peixoto, Mayla Regina Souza, Igor Ricardo Savoldi, Débora Ester Petry Marcelino, Mateus Tremea, Mônica Corrêa Ledur

**Affiliations:** 1 Programa de Pós-Graduação em Ciências Veterinárias, Universidade Estadual do Centro-Oeste, Guarapuava, Paraná, Brazil; 2 Embrapa Suínos e Aves, Concórdia, Santa Catarina, Brazil; 3 Programa de Pós-Graduação em Zootecnia, UDESC-Oeste, Chapecó, Santa Catarina, Brazil; 4 Faculdade de Concórdia—FACC, Concórdia, Santa Catarina, Brazil; 5 Universidade Federal de Santa Maria, campus Palmeira das Missões, Rio Grande do Sul, Brazil; USDA-Agricultural Research Service, UNITED STATES

## Abstract

The use of reference genes is required for relative quantification in gene expression analysis and the stability of these genes can be variable depending on the experimental design. Therefore, it is indispensable to test the reliability of endogenous genes previously to their use. This study evaluated nine candidate reference genes to select the most stable genes to be used as reference in gene expression studies with the femoral cartilage of normal and epiphysiolysis-affected broilers. The femur articular cartilage of 29 male broilers with 35 days of age was collected, frozen and further submitted to RNA extraction and quantitative PCR (qPCR) analysis. The candidate reference genes evaluated were *GAPDH*, *HMBS*, *HPRT1*, *MRPS27*, *MRPS30*, *RPL30*, *RPL4*, *RPL5*, *and RPLP1*. For the gene stability evaluation, three software were used: GeNorm, BestKeeper and NormFinder, and a global ranking was generated using the function RankAggreg. In this study, the *RPLP1* and *RPL5* were the most reliable endogenous genes being recommended for expression studies with femur cartilage in broilers with epiphysiolysis and possible other femur anomalies.

## Introduction

The use of gene expression analysis intends to clarify biological processes involved with several conditions in living organisms enabling the identification of diagnostic markers as therapeutic targets in the treatment of diseases [1]. The quantitative PCR (qPCR) is a fast, easy-to-use technique that provides simultaneous measurement of gene expression in many different samples for a limited number of genes [2]. In qPCR, fluorescent dyes are used to combine the amplification and detection steps of the PCR reaction in a single tube [3,4]. In addition, qPCR has been widely used for validating RNA-seq results due to its high sensitivity and precision [5]. When comparing to other techniques, it has advantages such as sensitivity, real-time detection of reaction progress, rapid results and accuracy in the measurement of the analyzed material [5,6]. Although the qPCR is a highly sensitive technique [7], its use must be standardized, especially considering the correct choice of reference genes to avoid mistaken results. The use of stable reference genes ensures the normalization in input RNA levels between samples, avoiding errors in the quantification [8]. Therefore, knowing the expression profile of these genes in each experimental design is crucial to obtain reliable results [7,9].

A valid reference gene should have its expression invariable between different experimental conditions, tissues or physiological state of the tissue or organism [8]. In relative quantification analyses, the use of reference genes is required to normalize the gene expression and to obtain the fold-change through mathematical algorithms [10–12]. Some of the most well-known reference genes are *GAPDH (glyceraldehyde 3-phosphate dehydrogenase)*, *PGK (phosphoglycerate kinase)*, *UBQ (ubiquitin)*, *RPL19 (ribosomal protein L19)*, *18S rRNA (ribosomal RNA 18S)*, *β-actin and β-tubulin* [7], which are used in several studies with many species. For instance, *GAPDH* and *18S rRNA* were used as individual reference genes for normalization of qPCR data in multiple studies in chickens [13–17]. However, the stability of reference genes can be altered depending on the tissue, age, treatment and other conditions, which makes it indispensable to test the stability of several genes before using those as reference [18–20]. The selection of stable reference genes is critical for the reliable performance of qPCR experiments [21].

The epiphysiolysis or femur head separation (FHS) consists of the separation of the growth plate from the articular cartilage [22]. This condition is a risk factor for infection and may cause bacterial chondronecrosis with osteomyelitis (BCO) in broilers [22,23]. BCO is also named as femur head necrosis (FHN) and occurs in the proximal femoral head, beginning with the degeneration of the articular cartilage and the growth plate. The bone degeneration begins due to bone deprotection, which may promote a high incidence of other locomotor pathologies, becoming a gateway for viruses and bacteria. Animals have serious problems with movement, affecting them unilaterally or bilaterally [24]. Epiphysiolysis may be an early stage of BCO, due to the fact that its pathogenesis is possibly initiated by damage to poorly mineralized chondrocyte columns (cartilage cells) in the epiphyseal and physeal growth plates of the leg bones, followed by colonization of osteochondritic clefts by opportunistic bacteria [25,26].

Hence, expression studies are required to clarify the genetic mechanism involved with femur pathologies. Although some reference genes were described for bones, it is important to elucidate those for the cartilage as well, since both tissues are involved in the development of those disorders. However, studies on candidate reference genes for chicken cartilage are not available to date. Therefore, to obtain stable genes to be used as reference in expression studies related to bone/cartilage disorders in broilers, the present study evaluated nine endogenous candidate genes in the articular cartilage of chickens with 35 days of age.

## Material and methods

### Animals and sample collection

The Embrapa Swine and Poultry National Research Center Ethical Committee of Animal Use (CEUA/CNPSA) approved this study under the protocol number 012/2012. The samples used were previously collected from chickens at 35 days of age, as described in detail by Peixoto et al. [27]. Briefly, 29 male broilers from a commercial line (14 normal and 15 lameness) were selected and sent to the Embrapa Swine and Poultry National Research Center, in Concórdia, SC, Brazil for sampling. The femur proximal head was classified based on the clinical examination of the separation of the growth plate (GP) from the articular cartilage (AC), according to Wideman et al. [25]. The normal (control) group (CG) was characterized by good adhesion between the GP and the AC, and the epiphysiolysis-affected group (AG) presented epiphysiolysis and, consequently, separation between the GP and AC. From the 29 broilers, the AC of eight normal (with average body weight of 2,336.75 ± 233.43 g) and 8 affected broilers (2,189.62 ± 444.23 g) were collected, stored in liquid nitrogen and transferred to a freezer at -80°C until the samples were processed. The sample collection for the CG was performed by removing the AC from the bone carefully using a scalpel at the moment of necropsy.

### RNA extraction

Eight samples of the AC from each group were ground in a mortar with liquid nitrogen. Then, Trizol reagent (Invitrogen, Carlsbad, CA, USA) was added for total RNA extraction, following the manufacturer's recommendations. To each 100mg of tissue, 1mL of Trizol was added and samples were vortexed and incubated for five minutes at room temperature. Then, 200μL of chloroform were added, the tubes were vigorously homogenized for 15 seconds and incubated at room temperature for five minutes. Centrifugation was performed at 16,000 xg at 4°C for fifteen minutes. The aqueous phase (containing the RNA) was separated into a new microtube, and 500μL of isopropanol were added, gently homogenized by inversion and incubated for 10 minutes at room temperature. Samples were centrifuged for 13,000 xg for 10 minutes at 4°C for RNA pellet formation. Subsequently, the supernatant was discarded and 1mL 75% Alcohol was added, homogenized and centrifuged at 10,500 xg for five minutes at 4°C. After discarding the supernatant, the remained pellet was dried for 15 to 20 minutes at room temperature and resuspended in 40μL of ultrapure water. To assure the samples quality and purity, the RNA clean up protocol was performed using the Qiagen Rneasy kit (Qiagen, Hilden, NRW, Germany), following the manufacturer´s instructions. The Biodrop (Biodrop, Cambridge, UK) spectrophotometer equipment was used to quantify the extracted samples, and samples with 260/280nm ratio above 1.8 were considered pure. To evaluate the integrity of each extracted sample, a 1.0% agarose gel was run and electrophoresed for 90 min.

### Complementary DNA (cDNA) synthesis

The cDNA synthesis was performed using the SuperScript III First-Strand Synthesis SuperMix kit (Invitrogen, Carlsbad, CA, USA), following the manufacturer's recommendations. Approximately 3 μg of total RNA were mixed with 1μL of 50μM OligoDT primer, and added 1 μL of Annealing buffer, completing the reaction up to 10μL. The samples were incubated in a thermocycler at 65°C for five minutes and chilled on ice for one minute. In sequence, 10 μL of the 2X First-Strand Reaction Enzyme Mix and 2μL of the SuperScript III/RNAseOUT Enzyme Mix (Invitrogen, Carlsbad, CA, USA) were added in the initial reaction for each sample. Subsequently, samples were incubated at 50°C for 50 minutes followed by 85°C for five minutes in the thermocycler. After, the samples were stored in a freezer -20°C for subsequent quantitative Polymerase Chain Reaction (qPCR).

### Real-time qPCR

In order to perform the qPCR analyses, primers for nine genes: *GAPDH*, *HMBS*, *HPRT1*, *MRPS27*, *MRPS30*, *RPL30*, *RPL4*, *RPL5* and *RPLP1* were designed using NCBI Primer-BLAST (Table 1). These genes were selected based on previous studies in chicken. For the quantification analyses, the reactions were prepared using 7.5 μL of Master Mix SYBR Green 2x, 0,166 μM of forward primer, 0,166 μM of reverse primer, 2 μl of cDNA at the 1:10 dilution and ultrapure water to complete 15 μL. The reactions were distributed in 96-well plates of Microamp Fast 96-Well Reaction Plate (Applied Biosystems, Foster City, CA, USA) and then submitted to the QuantStudio 6 Real-Time PCR equipment (Applied Biosystems, Foster City, CA, USA) with a temperature cycling of 95°C for 10 minutes, 40 cycles of 95°C for 15 seconds and 60°C for one minute. The melting curve was performed with the cycling of 95°C for 15 seconds, 60°C for 1 minute and 95°C for 15 seconds to evaluate the amplification specificities. Reactions were performed in duplicates to calculate the Ct (threshold cycle) mean, standard deviation and coefficient of variation between the two replicates.

**Table 1:** Primers for the candidate reference genes used for qPCR analysis in the femur articular cartilage of broilers.

Gene/ Ensembl ID	Functions	Primer Sequences (5'-3')
*HMBS*^1^ Hydroxymethylbilane synthase	Heme synthesis, porphyrin metabolism, Third enzyme of the biosynthetic pathway of the Hemegroup	F: ACTAGTTCACTTCGGCGAGC
*ENSGALG00000042939*		R: CTCAGGAGCTGACCTATGCG
*RPL5*^*2*^ Ribosomal Protein L5	Responsible for the synthesis of proteins in the cell, structural constituent of ribosome, 5S rRNA binding	F: AATATAACGCCTGATGGGATGG
*ENSGALG00000005922*		R: CTTGACTTCTCTCTTGGGTTTCT
*MRPS27*^2^ Mitochondrial Ribosomal Protein S27	Mitochondrial ribosome binding, rRNA binding, tRNA binding	F: GCTCCCAGCTCTATGGTTATG
*ENSGALG00000015002*		R: ATCACCTGCAAGGCTCTATTT
*MRPS30*^2^ Mitochondrial ribosomal protein S30	Structural constituent of ribosome, RNA binding.	F: CCTGAATCCCGAGGTTAACTATT
ENSGALG00000014874		R: GAGGTGCGGCTTATCATCTATC
*RPL4*^3^ Ribosomal Protein L4	poly(U) RNA binding, rRNA binding, structural constituent of ribosome	F: TGTTTGCCCCAACCAAGACT
*ENSGALG00000007711*		R: CTCCTCAATGCGGTGACCTT
*HPRT1*^4^ Hypoxanthine-guanine phosphoribosyltransferase	Purine synthesis in the salvage pathway	F: TGGGGATGACCTCTCAACCT
*ENSGALG00000006098*		R: TCCAACAAAGTCTGGCCGAT
*GAPDH*^4^ Glyceraldehyde-3-Phosphate Dehydrogenase	Transcription, RNA transport, DNA replication, and apoptosis.	F: TGGGAAGCTTACTGGAATGG
ENSGALG00000014442		R: ATCAGCAGCAGCCTTCACTAC
*RPLP1*^4^ Ribosomal protein lateral stalk subunit P1	Protein kinase activator activity, ribonucleoprotein complex binding, structural constituent of ribosome.	F: CCCTCATTCTCCACGACGACZ
*ENSGALG00000030878*		R: CCAGAGCCTTAGCAAAGAGAC
*RPL30*^3^ Ribosomal Protein L30	Antimicrobial humoral immune response mediated by an antimicrobial peptide, cytoplasmic translation, defense response to Gram-negative bacterium, the killing of cells of another organism.	F: ATGATTCGGCAAGGCAAAGC
*ENSGALG00000029897*		R: GTCAGAGTCACCTGGGTCAA

^1^Paludo et al. [34],

^2^Nascimento et al. [35],

^3^Petry et al. [36],

^4^Marciano et al. [37].

### Stability evaluation of the candidate reference genes

To evaluate the candidate reference genes from this study, three popular algorithms were used to identify the most stable expressed genes: geNorm [28], NormFinder [29] and BestKeeper [30]. The BestKeeper is an Excel-based software that classifies genes through an index (gene power) composed of Ct, fold-change (FC), standard deviation (SD) and coefficient of variation (CV) values [30]. The most consistent genes present SD values of Cts less than 1 and SD of FC less than 2. The authors do not recommend using genes with SD of Cts above 1.5 [30]. The GeNorm is a program that calculates a measurement of stability of the internal control gene (M) for each combination of two control genes tested, obtaining a transformed expression rate, and then calculates a standard deviation of those combinations of paired genes. From the lowest values of M, the two most stable genes are determined and values smaller than 1.5 indicate stable genes [28]. The NormFinder is a program that automatically calculates the stability value based on intra- and inter-group variation of the tested genes, considering their co-regulation, classifying genes according to their expression stability and similarity. Lower stability values indicate better or more stable genes to be used as normalizers. The authors suggested using data transformed into log_2_ [29]. When comparing with GeNorm, Bestkeeper has the advantage of using both constitutive and target genes in the analysis, while GeNorm analyzes only reference genes [30].

After performing each analysis, the stability values obtained in each tool were used to generate a ranking with the most stable genes using RankAggreg [31]. This is a package from the R environment that calculates the Spearman distance between two genes based on the Monte Carlo algorithm [32,33]. All of these analyses were performed using the endoGenes automatized pipeline available at https://github.com/hanielcedraz/endoGenes.

## Results

**Fig 1:**
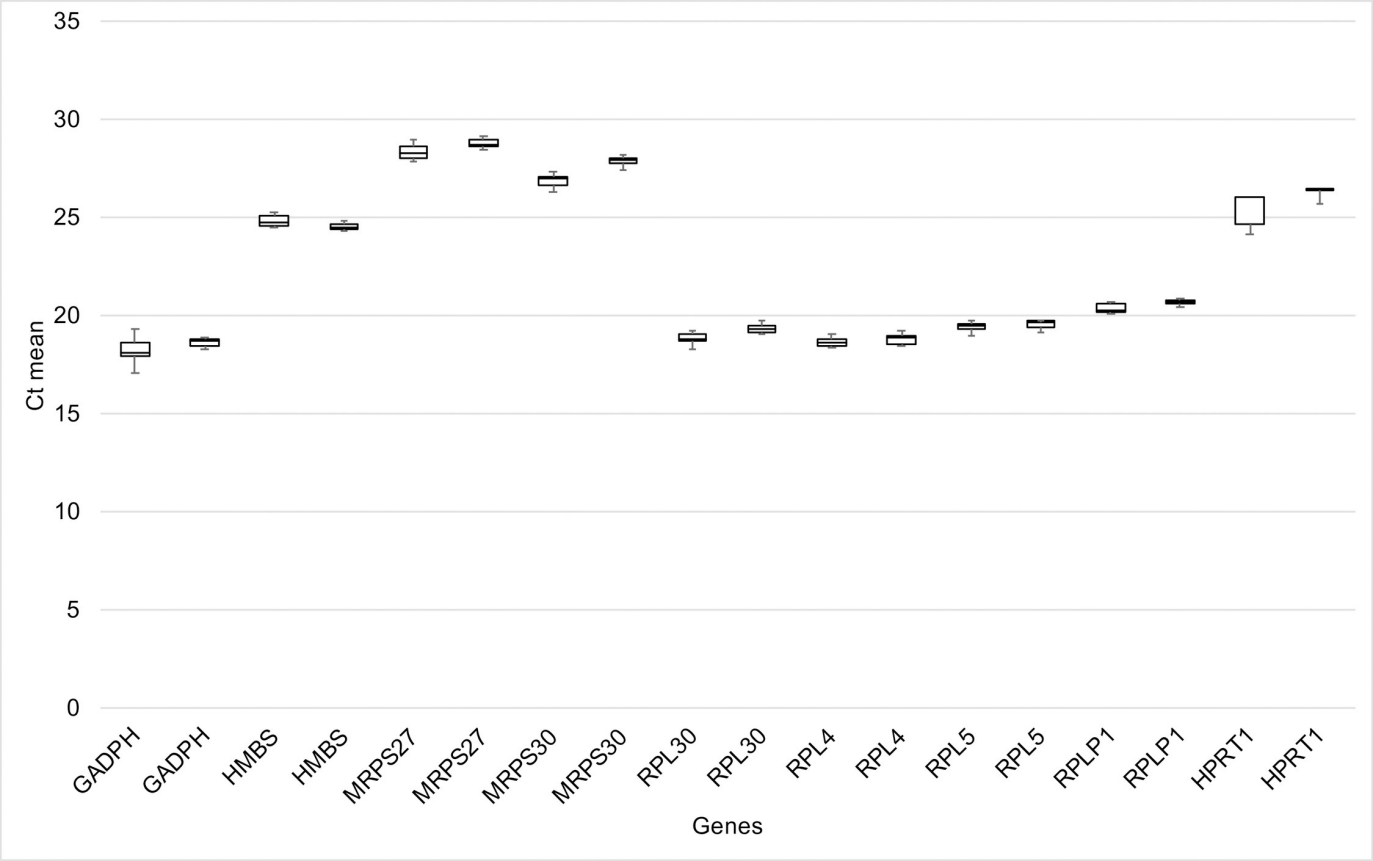
Cycle threshold (Ct) variation of the candidate reference gene in normal and proximal femoral epiphysiolysis-affected broilers. CG: control group and AG: affected group.

**Fig 2:**
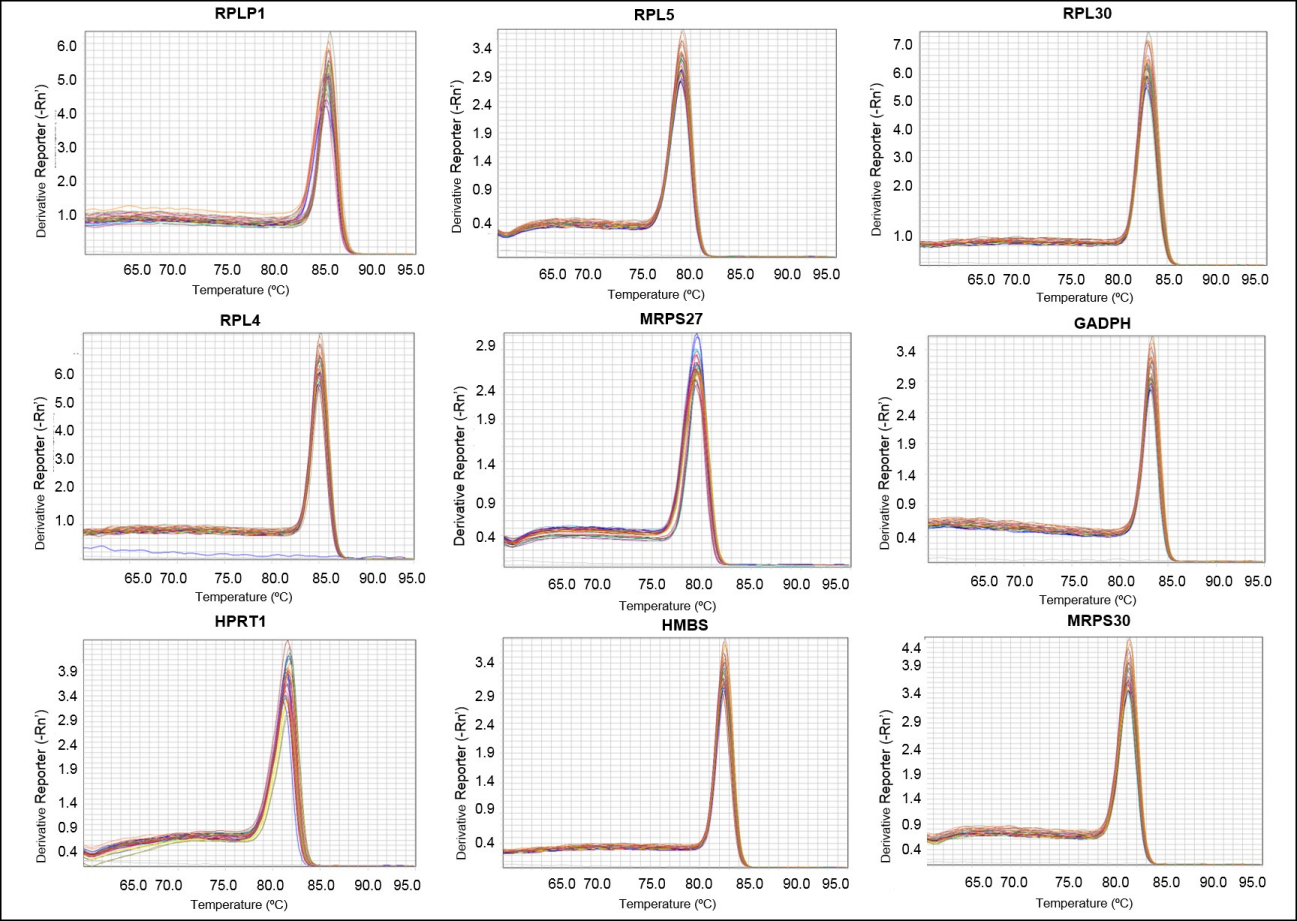
Melting curve for the 9 candidate reference genes in the femur articular cartilage of broilers for primers specificity evaluation.

The total RNA average concentration was 195.92 ng/μL for the normal and 172.01 ng/μL for the epiphysiolysis-affected broilers. Regarding RNA quality, the mean A260/280 ratio was 2.07 for normal and 2.08 for the affected chickens. These values demonstrate good quality of samples. The Ct mean (± SD) values of the candidate reference genes ranged from approximately 18.51 ± 0.65 to 28.68 ± 0.59 (Fig 1 and S1 Table). According to the melting curve analysis, all genes showed specific amplification (Fig 2).

**Fig 3:**
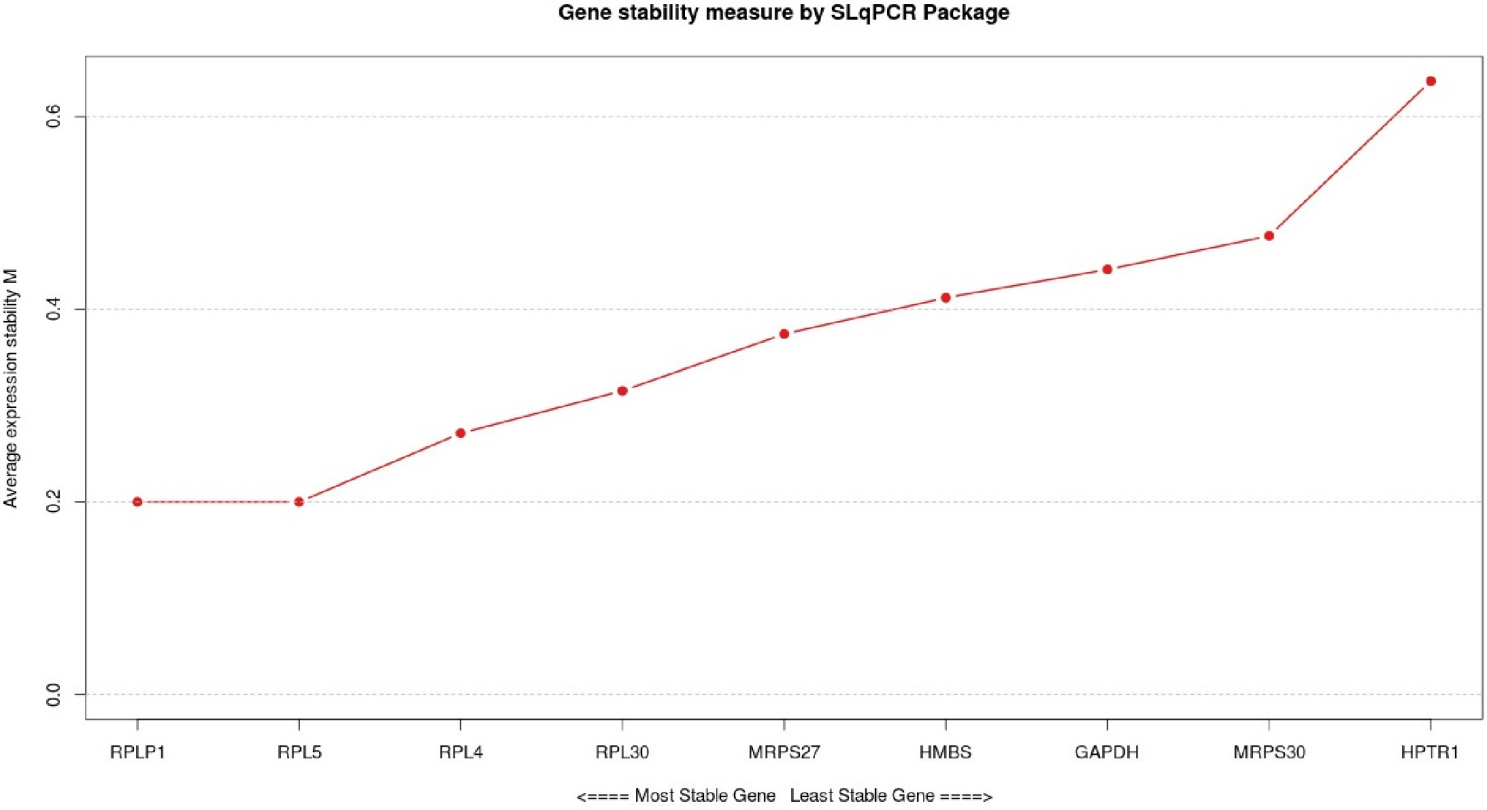
Ranking of candidate reference genes based on the average expression M stability value using the geNorm software.

**Fig 4:**
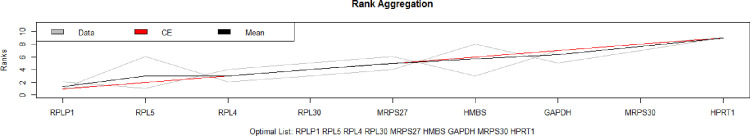
Candidate reference genes ranked with the Rankaggreg function based on the geNorm, BestKeeper and NormFinder stability values.

A similar expression profile among the *RPLP1*, *MRPS30* and *HPRT1* genes was obtained with the evaluated software (Fig 4 and Table 2). The genes *RPL5* and *RPLP1* were classified as the two most stable with the software BestKeeper (Table 2) and GeNorm (Fig 3 and Table 2), and *RPLP1* and *RPL4* with NormFinder (Table 2). The gene *RPL5* was classified in the sixth position with the software NormFinder (Table 2) differing from the Bestkeeper and GeNorm results. The least stable gene was *HPRT1* according to all the software evaluated (Figs 3 and 4 and Table 2). The gene *HMBS* had a divergent classification among BestKeeper, GeNorm and NormFinder, ranking this gene, respectively, in the third, sixth and eighth positions (Table 2).

**Table 2:** Gene classification values and ranking (in parenthesis) according to the three algorithms analyzed and the general rank generated with the RankAggreg.

Gene	Bestkeeper (SD of [±Ct])	NormFinder (S-value)	GeNorm (M-value)	RankAggreg (Ranking)
*RPLP1*	0.266 (2)	0.15 (1)	0.200 (1)	1
*RPL5*	0.239 (1)	0.22 (6)	0.200 (2)	2
*RPL4*	0.325 (4)	0.15 (2)	0.271 (3)	3
*RPL30*	0.367 (5)	0.16 (3)	0.315 (4)	4
*MRPS27*	0.433 (6)	0.17 (4)	0.374 (5)	5
*HMBS*	0.311 (3)	0.42 (8)	0.412 (6)	6
*GAPDH*	0.472 (7)	0.22 (5)	0.441 (7)	7
*MRPS30*	0.559 (8)	0.39 (7)	0.476 (8)	8
*HPRT1*	0.929 (9)	0.48 (9)	0.637 (9)	9

Since there were some variations in the classification of the reference genes in the results of each software separately, the RankAggreg was used to generate an overall classification, where *RPLP1* and *RPL5* genes were identified as the most stable ones (Fig 4 and Table 2). RankAggreg results were similar to those obtained with the evaluation of geNorm, BestKeeper and NormFinder regarding the *MRPS30* and *HPRT1* genes, which were classified as the least stable genes from those evaluated in our study (Figs 3 and 4 and Table 2).

## Discussion

The evaluation of endogenous genes stability is an essential step in relative quantification analyses of gene expression and needs to be performed previously to its use to avoid biased results. Due to the widespread use of qPCR technique, several protocols, reagents, and methods of analysis have emerged, which can lead to contradictory results [8,38]. In order to obtain high quality results, the samples must have sufficient quantity and integrity of the RNA, generating results with high precision, sensitivity, and reproducibility for posterior analysis [29,30,39]. Another important factor is the evaluation of a set of candidate reference genes, which is fundamental for obtaining reliable results from qPCR studies [30,39]. It should be noted that different conditions and experiments require different genes to be used as normalizers, needing a specific search for genes with stable expression patterns [8,39]. Here, total RNA with good quality was used for the further analysis, according to the MIQE recommendations [40]. Nine candidate reference genes were evaluated, considering stability parameters and consistency of expression with four different specific software (Table 2) widely used in similar studies [30,37,41]. Although the search for the best reference genes is needed, there is no standard methodology established for this purpose, and a wide variety of approaches is available. Another issue is that there is no standardization of the values used by the algorithms, with some having a clear stability threshold value, while others do not, and this value is relevant for checking the stability of the gene. Thus, the use of several software could lead to a better selection of the most reliable reference genes.

In chickens, studies evaluating gene stability in several tissues, such as muscle, embryo, ovary, uterus, lung and heart fibroblasts have been reported [35,37,42–45]. However, there are no studies regarding the stability of endogenous genes in poultry articular cartilage tissue. Therefore, our study is important to characterize this type of sample, especially because this tissue is involved in several bone disorders or locomotor problems in commercial broilers.

Variations in the gene stability classification, such as the *HMBS* that was ranked as 3^rd^, 8^th^ or 6^th^ (Fig 3 and Table 2), was observed depending on the software used. Nascimento et al. [35] also found variation in the results obtained by the software used to evaluate reference genes in *pectoralis major* muscle of broilers. These differences can be explained by the fact that the software have different algorithms, and have been developed to address different types of experiments. The BestKeeper allows comparing the expression level of up to ten endogenous genes, generating an index combining the evaluated genes, with a high sensitivity to genes with very different expression levels [30]. The NormFinder automatically calculates the stability value (S) of all endogenous genes tested on a set of samples, regardless the number of samples and groups, showing a high sensitivity to co-regulating genes [29]. On the other hand, geNorm does not consider co-expression of candidate reference genes and classifies the two most stable reference genes among those tested [28].

Although there is a great variation in the classification of genes by different software, there is no recommendation for the best method of gene selection, nor a pattern that indicates good or poor stability [46]. After the analysis with BestKeeper, geNorm and NormFinder, the geNorm seemed to be the most suitable software to choose the most reliable genes, because its results were more similar to those found with RankAggreg. Through a general classification obtained with the RankAggreg function, *RPLP1* and *RPL5* were the most stable genes found in our study (Fig 4 and Table 2). Regarding the genes stability, those best classified in the general ranking also had a good stability in each software separately, since they had values within the parameters suggested by each software: S <0.5, M <1.5 and [± Ct] SD <1, for the NormFinder, GeNorm and Bestkeeper, respectively (Fig 3 and Table 2). The use of various tools to choose the reference genes allowed a broad check of the variation of the expression of these genes. Although RankAggreg provides a general classification of genes, it does not mean that all genes are stable or vice-versa [39]. The evaluation of the output from each software is needed to confirm whether the genes are indeed stable or not [8,39], as previously shown.

Several genes have been reported as reference genes. Ribosome proteins are suggested as good endogenous genes, because of their role in the production of ribosomes [47–49], since they are important components of the basic physiological processes in all cells [49]. Here, several known endogenous genes, such as *GAPDH*, *HMBS* and *HPRT1*, as well as many candidate ribosomal proteins (*RPLs* and *MRPLs*) were evaluated for their stability. The *HPRT1* was the least stable gene, while *RPLP1* was one of the most stable. These two genes were unstable at different stages of cardiac development in rats and were not indicated to be used as reference genes in cardiac tissue [50]. However, Nakayama et al. [51] and Nascimento et al. [35] found similar results from our study, where the *RPLP1* was suitable for normalization of gene expression in nasal tissue in humans and muscle tissue in broilers, respectively. Furthermore, some studies that evaluated the stability of the *HMBS* gene found this gene to be one of the most stable when considering several tissues [34,35,49,52–54]. Zhang et al. [52], for example, evaluating the stability of eight reference genes in 10 types of Boer goat tissues found that the *HMBS* gene was the third most stable and therefore recommended this gene for calibrating gene expression analyses of goat tissues from this breed by real-time qPCR. In broiler chickens *pectoralis* major muscle, the *HMBS* gene was also found to be the most stable by Nascimento et al. [35]. According to these authors, the *HMBS* and *HPRT1* genes were the most stable and could be used to normalize expression data in the *pectoralis major* muscle of chickens submitted to heat stress [51]. Zhang et al. [53] showed that *HMBS* was the most suitable gene for chickens gut, while Paludo et al. [34] used *HMBS* as an endogenous gene to study bones from broilers with 45 days of age affected with femoral head necrosis. This gene has been used as endogenous in many species, in different tissues and ages [34,49,54]. Here, the *HMBS* gene had a good classification in the Bestkeeper tool, but not in NormFinder and GeNorm (Table 2). These results reinforce that gene stability could be influenced by different factors, such as age, tissue and conditions [35,49]. Furthermore, the *HPRT1* gene was classified as the least stable gene by the three software, although this gene has already been considered a good reference gene for different swine tissues [54] and mice [55], and is widely used in rats as a reference gene in qPCR studies.

The nuclear genes *MRPS27* and *MRPS30* encoding mitochondrial ribosomal proteins have high activity in muscle tissues and in the synthesis of proteins within the mitochondria [56]. In quail, the most stable genes evaluated in several tissues were *MRPS30*, *EEF1* and *HMBS* in the thigh muscle, *B2M*, *UBC* and *GAPDH* in the brain, *MRPS30*, *TFRC* and *HMBS* in the heart, and *EEF1*, *LDHA* and *HMBS* in the spleen [57]. Furthermore, these authors also recommended testing the expression of endogenous genes that could vary between male and female quails [57]. When evaluating the muscle tissue of chickens under heat stress, the genes *MRPS27*, *RPL5* and *MRPS30* were considered stable according to the general classification of RankAggreg and can be used as normalizers in qPCR analysis of target genes in this condition [49].

Ribosomal proteins are crucial to the development and tissue homeostasis [58]. Wang et al. [59] suggest ribosomal proteins as good candidates to substitute the traditional reference genes as internal controls in real-time PCR assays. Previously, ribosomal proteins were recommended only for less sensitive detection methods like Northern blot [60]. However, recent studies evaluating the stability of reference genes have reported outstanding stabilities of ribosomal proteins in different cell lines and tissues of mammals [61–63], fish [64], shellfish [65] and plants [66]. The gene *RPLP1* was studied as a reference gene in several animal species, such as *Homo sapiens* [58], *Mus musculus* [67], *Rattus norvegicus* [68,69], *Gallus gallus* [43,70], *Bos Taurus* [71] and *Anopheles gambiae* [72], having a role in the elongation step of protein synthesis [73]. This gene had a divergent stability ranking between neonatal and cardiosphere-derived adult cells, indicating that its gene expression was age-dependent [50]. *RPLP1* expression seems to be tissue-dependent, because it was the least stable gene in breast muscle of chickens [37] while it was the most stable gene in the current study. Benak et al. (2019) [74], when selecting optimal reference genes for gene expression studies in chronically hypoxic rat heart, found that the gene *RPLP1* was one of the most stable genes in the left and right ventricle, similarly to our study. This gene has a variable expression according to the tissue, age and species used in the experiment, reinforcing the importance of checking reference genes even when similar issues have already been addressed.

The *RPL5*, a gene that encodes a small protein that is a component of the 60S subunit and is responsible for transporting the *5S rRNA* to the nucleolus, was the 2^nd^ most stable gene in the general ranking in the current study. Our results corroborate with Oliveira et al. [49], which evaluated the *RPL5* gene in chicken muscle tissue of males and females, and was indicated as suitable for normalization of gene expression. The RPL5 is a constitutive protein in the large ribosomal subunit that catalyzes mRNA-directed protein synthesis [60]. When evaluating assays of the cornea in various murine disease models, Ren et al. [55] recommended *RPL5* as a reference gene, showing stability in that study. *RPL5* was also a stable gene when studying heart failure of the right ventricles in humans [75] and tissues of red abalone *Haliotis rufescens* (Mollusca, Vetigastropoda) [59,76]. Kim et al. [77] used *RPL5* as a reference gene to calibrate the reverse-transcribed cDNA templates for samples of cerebral ganglion, pleuro-pedal ganglion, ovary, gills, intestine and adductor muscle of Pacific abalone (*Haliotis discus hannai*). Marciano et al. [37], when studying breast muscle of chicken also found *RPL5* as one of the most stable genes. These results corroborate with those found in our study, showing that this gene is suitable as normalizer for several species, including chickens.

In the current study, we showed that *RPLP1* and *RPL5* were ranked as the most stable genes when femur head’s articular cartilage of broilers were evaluated, contributing to the understanding of gene expression profiles of candidate endogenous genes in chickens. These results can also help to clarify the etiology of bone-related problems in avian and other species. Despite the indication of using two or more reference genes in gene expression studies, it is common to find the use of only one gene, and based merely in the literature, not following the MIQE recommendations [39,40]. Therefore, in order to obtain the best reference genes, it is necessary to evaluate a broad panel of genes and software, considering the complexity of the experimental designs and tissues [39]. Thus, the choise of the most reliable reference genes for relative quantification analysis reduces the selection of false endogenous genes, improving the accuracy of the results [78].

## Conclusions

The *RPL5* and *RPLP1* were the most reliable endogenous genes for qPCR analyses in the femur articular cartilage tissue of normal and epiphysiolysis-affected broilers. This is the first study evaluating reference genes in the chicken articular cartilage. Our results can be useful for investigating articular disorders in chickens and other species by the analysis of articular cartilage gene expression.

## Supporting information

S1 Table Ct means for the nine candidate reference genes. (DOCX)
