# Supplementary material for: Reference genes for proximal femoral epiphysiolysis expression studies in broilers cartilage
Source: PLoS One. 2020 Aug 25;15(8):e0238189. doi: 10.1371/journal.pone.0238189 (PMC7447007; doi:10.1371/journal.pone.0238189)
Supplement: S1 Table — (DOCX) [file pone.0238189.s001.docx]

**S1 Table. Ct means for the nine candidate reference genes.**

|  |  | **Ct Mean ± SD** |  |
| --- | --- | --- | --- |
| **Gene** | **CG** | **AG** | **General mean** |
| *RPLP1* | 20.36 ± 0.25 | 20.77 ± 0.39 | 20.56 ± 0.32 |
| *RPL5* | 19.45 ± 0.23 | 19.64 ± 0.44 | 19.54 ± 0.33 |
| *RPL4* | 18.66 ± 0.25 | 18.99 ± 0.68 | 18.82 ± 0.46 |
| *RPL30* | 18.82 ±0.31 | 19.50 ± 0.56 | 19.16 ± 0.43 |
| *MRPS27* | 28.44 ± 0.63 | 28.90 ± 0.47 | 28.67 ± 0.55 |
| GAPDH | 18.25 ± 0.70 | 18.77 ± 0.52 | 18.51 ± 0.61 |
| *HMBS* | 24.82 ± 0.30 | 24.60 ± 0.48 | 24.71 ± 0.39 |
| *MRPS30* | 26.88 ± 0.34 | 27.98 ± 0.50 | 27.43 ± 0.42 |
| *HPRT1* | 25.90 ± 1.44 | 26.77 ± 0.96 | 26.33 ± 1.20 |

CG= control group; AG= affected group.
